# The *Pseudomonas* Quinolone Signal (PQS): Not Just for Quorum Sensing Anymore

**DOI:** 10.3389/fcimb.2018.00230

**Published:** 2018-07-04

**Authors:** Jinshui Lin, Juanli Cheng, Yao Wang, Xihui Shen

**Affiliations:** ^1^Shaanxi Engineering and Technological Research Center for Conservation and Utilization of Regional Biological Resources, Yan'an University, Yan'an, China; ^2^College of Life Sciences, Yan'an University, Yan'an, China; ^3^Shaanxi Key Laboratory of Agricultural and Environmental Microbiology, College of Life Sciences, Northwest A&F University, Yangling, China; ^4^State Key Laboratory of Crop Stress Biology for Arid Areas, Northwest A&F University, Yangling, China

**Keywords:** *Pseudomonas aeruginosa*, PQS, quorum sensing, iron acquisition, cytotoxicity, outer-membrane vesicles, immune regulation

## Abstract

The *Pseudomonas* quinolone signal (PQS) has been studied primarily in the context of its role as a quorum-sensing signaling molecule. Recent data suggest, however, that this molecule may also function to mediate iron acquisition, cytotoxicity, outer-membrane vesicle biogenesis, or to exert host immune modulatory activities.

## Introduction

*Pseudomonas aeruginosa* is a gram-negative opportunistic pathogen that is the cause of a broad range of human diseases, such as septicemia, pneumonia, and other critical infections (Bleves et al., 2010; Lin et al., 2015; Valentini et al., 2017). The pathogenesis of *P. aeruginosa* is controlled by several bacterial virulence factors that regulate adhesion and/or interrupt host cell signaling pathways (Valentini et al., 2017). *P. aeruginosa* has an armory of cell-associated (flagella, pili, lectins, alginate/biofilm, and lipopolysaccharide) and extracellular (proteases, hemolysins, cytotoxin, pyocyanin, siderophores, exotoxin A, exoenzyme S, and exoenzyme U) virulence factors (Strateva and Mitov, 2011).

The generation of some *P. aeruginosa* virulence factors is organized by a cell density monitoring mechanism called quorum sensing (QS) (Strateva and Mitov, 2011). Usually QS bacteria generate and liberate small chemical signals, and at high population densities, the gathered signals interface with cognate receptors to prompt the expression of numerous target genes, including genes that encode the generation of virulence factors (Lee and Zhang, 2015). *P. aeruginosa* has four QS systems that function independently and dependently, namely, the Las, Rhl, and quinolone-based QS systems and the recently determined IQS-dependent system (Lee and Zhang, 2015). The Las and Rhl systems are controlled via the acyl-homoserine lactone (AHL) autoinducers N-(3-oxododecanoyl)-L-homoserine lactone (OdDHL) and N-butyryl-L-homoserine lactone (BHL), respectively. LasR and RhlR (the QS receptors for the Las and Rhl systems, respectively) homodimerize after binding their signal molecules, which allows them to connect to conserved *las-rhl* boxes in the promoters of target genes, thus prompting their transcription (Lee and Zhang, 2015). The quinolone-based QS system acts mainly via 2-heptyl-3-hydroxy-4-quinolone, called the *Pseudomonas* quinolone signal (PQS), which connects the LysR-type transcriptional regulator PqsR to stimulate some virulence genes (Lee and Zhang, 2015). These signaling systems create a global regulatory network and are believed to regulate the expression of up to 12% of the *P. aeruginosa* genome (Schuster et al., 2003; Wagner et al., 2003; Déziel et al., 2005; Schuster and Greenberg, 2006).

A current evaluation revealed that PQS is obviously a multi-functional molecule that functions via several PqsR-dependent and PqsR-independent pathways (Rampioni et al., 2016). In addition, PQS not only affects cells by altering the transcriptional profiles of genes, but also binds directly to hundreds of previously unrecognized protein partners in the cell. These observations are the first to demonstrate that PQS may directly interact with several key virulence pathways (Hodgkinson et al., 2016; Baker et al., 2017; Dandela et al., 2018). Despite the great efforts made in PQS research, both *in vitro* and *in vivo*, its precise role remains poorly understood. In the present article, we stress the significance of the recently determined flexibility of PQS instead of the explicit mechanisms of the PQS QS system.

## PQS mediates QS

PQS was purified and determined in 1999 by Pesci and colleagues when they noted that the culture supernatant from wild-type *P. aeruginosa* PAO1 cells resulted in the obvious induction of a *lasB*′*-lacZ* reporter construct in a *lasR* mutant, which just about inactivated the *las* and *rhl* signaling pathways, and could not be imitated by OdDHL or BHL (Pesci et al., 1999). PQS is an alkylquinolone (2-heptyl-3-hydroxy-4-quinolone) that is chemically differentiated from the AHL signals of the *las* and *rhl* systems (Pesci et al., 1999).

**Figure 1:**
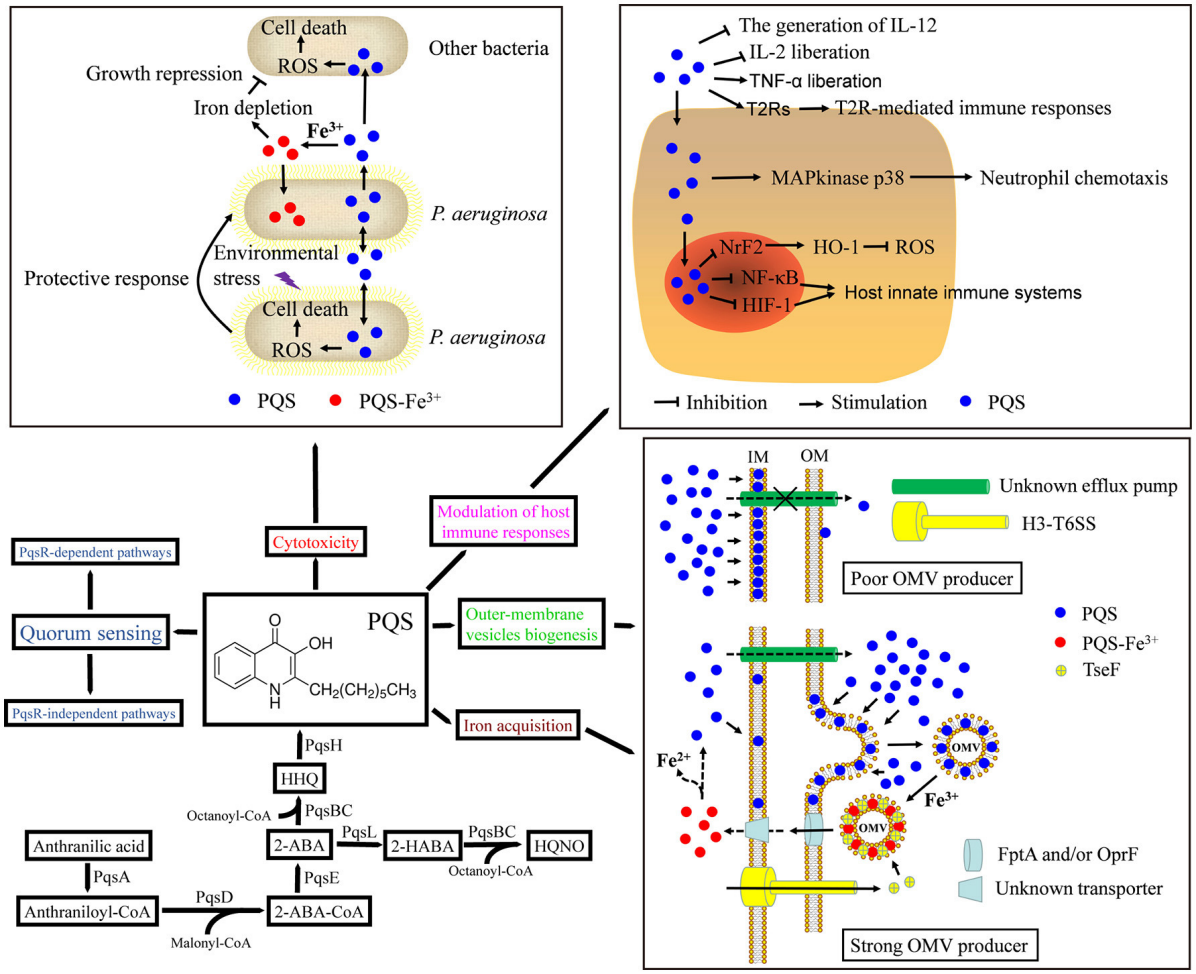
Biosynthetic pathway and multifunctionality of the *Pseudomonas* quinolone signal (PQS). Biosynthesis of HHQ requires PqsABCDE proteins. The mono-oxygenase PqsH then converts HHQ to PQS. PQS has been implicated in quorum sensing, iron acquisition, cytotoxicity, outer-membrane vesicle biogenesis, and modulation of the host immune response. See text for details. ROS, reactive oxygen species; IL-12, interleukin-12; IL-2, interleukin-2; TNF-α, tumor necrosis factor-α; T2Rs, taste family 2 bitter receptor proteins; NrF2, transcription factor NrF2; HO-1, heme oxygenase-1; NF-κB, nuclear transcription factor-κB; HIF-1, hypoxia-inducible factor 1; OM, outer membrane; IM, inner membrane; OMV, outer membrane vesicle; the dotted lines indicate a hypothesis. A second mono-oxygenase, PqsL, is required together with the *pqsABCDE* gene products for the synthesis of HQNO. Intermediates and products of the alkylquinolone biosynthetic pathway: 2-ABA-CoA, 2-aminobenzoylacetyl-coenzyme A; 2-ABA, 2-aminobenzoylacetate; 2-HABA, 2-hydroxylaminobenzoylacetate, HHQ, 2-heptyl-4-quinolone; HQNO, 4-hydroxy-2-heptylquinoline-N-oxide; PQS, 2-heptyl-3-hydroxy-4-quinolone.

The PQS synthesis cluster has been revealed to be made up of *pqsABCDE, phnAB*, and *pqsH* (Gallagher et al., 2002). In *P. aeruginosa* PAO1, these chromosomal genes consist of an operon made up of *pqsABCDE* (PA0996-PA1000), which is nearby *phnAB* (PA1001-PA1002) (Heeb et al., 2011). The *pqsH* (PA2587) gene is found somewhere else on the chromosome (Heeb et al., 2011). PqsA is an anthranilate-coenzyme A ligase (Gallagher et al., 2002; Coleman et al., 2008) that prompts anthranilate to produce anthraniloyl-coenzyme A, starting the first step in PQS biosynthesis. PqsB and PqsC, which create a secure heterodimer, as well as PqsD, are part of the FabH (β-ketoacyl-(acyl carrier protein) synthase III) family (Bredenbruch et al., 2005; Zhang et al., 2008; Bera et al., 2009; Dulcey et al., 2013; Drees et al., 2016). PqsD regulates the synthesis of 2-aminobenzoylacetate (2-ABA) from anthraniloyl-coenzyme A and malonyl-coenzyme A, and then PqsBC catalyzes the condensation of octanoyl-coenzyme A and 2-ABA to produce 2-heptyl-4-quinolone (HHQ) (Dulcey et al., 2013; Drees et al., 2016). The *pqsA, pqsB* and *pqsD* mutants do not produce alkylquinolones (AQs) (Diggle et al., 2003; Zhang et al., 2008). HHQ comes before PQS and can be transferred intercellularly among *P. aeruginosa* cells (Dubern and Diggle, 2008). The *pqsH* gene encodes a reported FAD-dependent mono-oxygenase necessary for the transformation of HHQ into PQS and supposedly hydroxylates HHQ at the 3-position (Figure 1; Gallagher et al., 2002; Déziel et al., 2004; Dubern and Diggle, 2008; Schertzer et al., 2009). Thus, the *pqsH* mutant does not produce PQS, but produces other AQs (Déziel et al., 2004). The transcription of *pqsH* is regulated by LasR, thus connecting PQS synthesis with the AHL-dependent *las* signaling pathway (Schertzer et al., 2009). A second mono-oxygenase, PqsL, uses reduced flavin to convert 2-ABA to 2-hydroxylaminobenzoylacetate (2-HABA), which is condensed with octanoyl-coenzyme A to form 4-hydroxy-2-heptylquinoline-N-oxide (HQNO) in a reaction catalyzed by PqsBC (Figure 1; Lepine et al., 2004; Drees et al., 2018). Mutants in *pqsL* cause the overgeneration of PQS, indicating that AQ biosynthetic intermediates are moved in these mutants to generate PQS (D'Argenio et al., 2002; Déziel et al., 2004; Lepine et al., 2004). Besides PqsABCD, PqsE has a part in HHQ synthesis (Drees and Fetzner, 2015). PqsE functions as a thioesterase in alkylquinolone biosynthesis, hydrolyzing the biosynthetic intermediate 2-aminobenzoylacetyl-coenzyme A to create 2-ABA, the predecessor of HHQ and 2-aminoacetophenone (Drees and Fetzner, 2015). The part of PqsE can be partially counteracted by the broad-specificity thioesterase TesB, which reveals why *pqsE* deletion mutants continue to synthesize HHQ and PQS (Figure 1; Drees and Fetzner, 2015). PqsE functions as a pathway-specific thioesterase, but it also adds to the control of bacterial virulence through an unidentified mechanism; its enzymatic activity might not be in charge of its regulatory activity (Zender et al., 2016).

Just following the determination of the PQS signal, the receptor PqsR (then referred to as MvfR) was involved in the control of PQS production (Cao et al., 2001). PqsR is a LysR-type transcriptional regulator that links to the promoter area of the *pqsABCDE* operon and immediately regulates its expression to produce an auto-regulatory loop (Cao et al., 2001; Gallagher et al., 2002; McGrath et al., 2004; Wade et al., 2005; Xiao et al., 2006). The expression of *pqsR* is then regulated by the AHL-dependent QS regulator, LasR (Camilli and Bassler, 2006). PqsR has two ligands, HHQ and PQS, which prompt PqsR linking to the *pqsABCDE* promoter region (Xiao et al., 2006). PQS binds PqsR with higher avidity than HHQ, and PQS is about 100-fold more active than HHQ (Wade et al., 2005; Xiao et al., 2006). The mutation of *pqsR* ends up in lowered pyocyanin, elastase, exoprotein, and 3-oxo-C12-HSL generation, termination of *phnAB* and *pqsABCDE* expression, and AQ biosynthesis, along with being impaired so it cannot induce disease in plants and animals (Cao et al., 2001; Gallagher et al., 2002; von Bodman et al., 2008; Schertzer et al., 2009). This suggests that PqsR is necessary for PQS signal transduction.

The part of the *pqs* signaling system in promoting infection and controlling virulence factors has been evaluated in numerous studies. Null mutants of the *pqs* system show lowered biofilm development and reduced generation of virulence factors, including pyocyanin, elastase, lectin, and rhamnolipids (Rahme et al., 1997, 2000; Cao et al., 2001; Diggle et al., 2003; Bala et al., 2015). The *pqs* system is also required for complete *P. aeruginosa* virulence toward plants (Cao et al., 2001), nematodes (Gallagher et al., 2002), and mice (Cao et al., 2001; Lau et al., 2004). In addition, PQS has been identified in the sputum of cystic fibrosis patients infected with *P. aeruginosa* (Collier et al., 2002; Abdalla et al., 2017). Nevertheless, the part PQS plays in the modulation of virulence gene expression is currently up for discussion. In a mouse burn infection model, the *pqsA* mutant displays greatly reduced virulence compared to the wild type strain (Déziel et al., 2005; Xiao et al., 2006). It is interesting to note that the *pqsH* mutant is as harmful as the wild-type in the identical mouse burn infection model (Xiao et al., 2006), but it has reduced virulence in nematodes (Gallagher et al., 2002). There are also contradictory evaluations on the efficacy of PQS in prompting the *pqsA* promoter via PqsR. One evaluation discovered that in strain PA14, PQS is more beneficial than HHQ at upregulating *pqsA* (Xiao et al., 2006). Nevertheless, a different evaluation showed that in strain PAO1, PQS has less of an impact (Fletcher et al., 2007). In addition, PQS has been documented to control the expression of 182 genes, of which a large number are PqsR-independent (Rampioni et al., 2016). Altogether, PQS may not have as pivotal of a part in the immediate control of virulence factors in *P. aeruginosa* as was initially believed and it might have emerged as an unexpected byproduct with other functions (Bredenbruch et al., 2006; Diggle et al., 2007).

## PQS mediates iron acquisition

As well as having a part as a QS signaling molecule, PQS chelates ferric iron (Fe^3+^) in a similar fashion to the quinolobactin siderophore from *Pseudomonas fluorescens* (Mossialos et al., 2000; Bredenbruch et al., 2006). PQS has high affinity for iron and can prompt the expression of genes for the biosynthesis of the siderophores pyoverdine and pyochelin (Bredenbruch et al., 2006; Diggle et al., 2007; Bala et al., 2015; Popat et al., 2017). One reason for this is that PQS treatment imitates iron starvation, as the PQS traps iron in a non-deliverable manner (Diggle et al., 2007; Popat et al., 2017). About 60% of the PQS generated by *P. aeruginosa* is connected to the cell envelope (Lepine et al., 2003; Diggle et al., 2007), and the membranes of stationary-phase LB-grown *P. aeruginosa* are noticeably pink because of complexed Fe^3+^, potentially because of insoluble PQS-iron complexes (Royt et al., 2001, 2007). This has been hypothesized to act in retaining free Fe^3+^, which can then be taken up by siderophores to more efficiently scavenge accessible iron under iron-deficient conditions (Diggle et al., 2007). While this is an elaborate clarification, it is likely insufficient (Diggle et al., 2007). For instance, Rampioni *et al*. revealed that the expression of siderophore-associated genes in *P. aeruginosa* is also intensely PqsE-dependent, suggesting that iron trapping by PQS by itself does not completely control siderophore genes (Rampioni et al., 2010). Hazan et al. documented that iron counterbalances PQS-dependent control by “fine-tuning” its activity, potentially by lowering PQS activity when complexed with it (Hazan et al., 2010). Further, experiments conducted in a *P. aeruginosa pvdD*/*pchEF* double mutant, which is not able to generate the siderophores pyoverdine and pyochelin, demonstrated that growth is escalated in iron-deficient media via the addition of PQS-Fe^3+^ in a fashion like that induced by FeCl_3_ (Diggle et al., 2007). *P. aeruginosa* also expresses heme, ferrous iron, and ferric citrate uptake systems, any of which could mediate iron uptake depending on the growth conditions (Schalk and Cunrath, 2016). Furthermore, the newly identified pseudopaline metallophore could potentially mediate uptake of ferrous iron from PQS-Fe^3+^ complexes so long as a reductant (such as phenazines or media components) is present (Lhospice et al., 2017; Mastropasqua et al., 2017). However, the point is taken that PQS-Fe^3+^ is likely not inducing growth simply by promoting siderophore-dependent uptake. Thus, PQS may function as an iron trap and storage molecule in cell membranes, and it could transport iron immediately to cells.

Recently, we reported that PQS-associated iron is utilized by *P. aeruginosa* PAO1 in a TseF-mediated process (Figure 1). TseF is secreted by the Type VI Secretion System (T6SS) and associates with PQS, as well as the receptors OprF and FptA. PQS also associates with outer-membrane vesicles (OMVs), contributing to the sequestration of iron ions in OMVs, which can then be utilized by *P. aeruginosa* under iron-limited conditions. Then, TseF enables the delivery of OMV-associated PQS-Fe^3+^ to bacterial cells by involving the Fe(III)-pyochelin receptor FptA and the porin OprF. However, this iron uptake pathway appears to have low efficiency (Lin et al., 2017). Thus, PQS provides a means of scavenging and concentrating freely diffusible iron ions and delivering them directly to the cells or to other siderophores.

## PQS mediates cytotoxicity

The 4-quinolone family, which includes PQS, has been acknowledged for the antimicrobial activity of numerous members (Heeb et al., 2011). Accordingly, PQS was found to have concentration-dependent toxic effects (Diggle et al., 2007; Haussler and Becker, 2008). Exogenously added PQS extends the lag phase and decreases the growth rate of *P. aeruginosa* under aerobic conditions in either iron-sufficient or iron-deficient medium (Diggle et al., 2003, 2007; Haussler and Becker, 2008; Toyofuku et al., 2008). PQS suppresses the growth of some species in addition to *P. aeruginosa*, such as gram-negative and gram-positive bacteria (Toyofuku et al., 2010). The impact of PQS on bacterial growth is unlike that of antibiotics, which function in a bacteriostatic or bacteriocidal manner, but rather induces the bacteria to growth more slowly (Toyofuku et al., 2010). The possible underlying mechanism is that PQS may deplete iron from the medium or induce oxidative stress (Figure 1). A previous study demonstrated that the growth of a *P. aeruginosa* mutant that does not produce siderophores is greatly repressed, compared to the wild-type strain, in iron-deficient media by adding PQS, and that growth is restored upon provision of PQS-Fe^3+^ or FeCl_3_ (Diggle et al., 2007). Not long ago, Toyofuku *et al*. showed that growth repression by PQS is impeded by adding iron to the medium, suggesting that iron-chelating activity could be involved (Toyofuku et al., 2010). These data indicate that iron availability has a pivotal part in growth inhibition, likely because of the iron-chelating activity of PQS. Likewise, iron lowers the clinical efficacy of some drugs, including tetracycline, via the generation of iron-drug complexes (Avery et al., 2004). Therefore, iron may hinder the impact of PQS by establishing a PQS-Fe^3+^ precipitate. At this point, it is hard to establish if adding iron reinstates lowered levels in the medium or reverses PQS-mediated growth impediment because of precipitation (Toyofuku et al., 2010). Toyofuku et al. reported that no correlation was observed between bacterial strains whose growth was repressed by PQS and siderophore-producing bacteria, which goes against the hypothesis that siderophore production determines susceptibility to PQS (Toyofuku et al., 2010). Therefore, other factors may be responsible for susceptibility to PQS.

A frequent cell-killing mechanism of antimicrobials pertains to the generation of hydroxyl radicals (∙OH) (Kohanski et al., 2008; Dwyer et al., 2009), and PQS can induce the generation of reactive oxygen species (ROS) and resultant toxicity in *P. aeruginosa* (Haussler and Becker, 2008; Nguyen et al., 2011; Pezzoni et al., 2015). PQS can prompt oxidative stress in macrophages and lung epithelial cell lines (Abdalla et al., 2017). The red-colored PQS–Fe^3+^ complex bestows the “red death” lethal phenotype to *P. aeruginosa* in a *Caenorhabditis elegans* infection model (Zaborin et al., 2009). At the same time, PQS sensitizes *P. aeruginosa* to the effects of ultraviolet-A (UVA) radiation, possibly acting as an endogenous photosensitizer (Pezzoni et al., 2015). Absorption of UVA by PQS leads to its own photo-degradation and the production of singlet oxygen and superoxide anions, indicating that it raises oxidative damage to biological targets on UVA exposure and bestows high sensitivity to UVA in contrast to enteric bacteria (Pezzoni et al., 2015). Further, the pro-oxidant activity of PQS raises the sensitivity of *P. aeruginosa* to peroxide and different antibiotics (Haussler and Becker, 2008; Nguyen et al., 2011), potentially causing cell lysis and DNA release. PQS can prompt autolysis in *P. aeruginosa*, specifically if overexpressed (D'Argenio et al., 2002), and regularly adding PQS to *P. aeruginosa* prompts genes in charge of the oxidative stress response (Bredenbruch et al., 2006). In addition, PQS has been indicated to balance growth and death in *P. aeruginosa* populations by prompting a protective reaction in some cells while eradicating others (Haussler and Becker, 2008). Therefore, PQS's inhibitory impact on bacterial growth may modulate the growth of bacterial communities and bestow a survival edge when *P. aeruginosa* is developing with rival microorganisms.

## PQS mediates OMV biogenesis

AQs are lipophilic molecules with poor aqueous solubility and PQS is more hydrophobic than HHQ. PQS has a solubility of about 1 mg/L (~5 μM) in water (Lepine et al., 2003; Mashburn and Whiteley, 2005). Nevertheless, *P. aeruginosa* appears to have developed a more direct solution to the problem of PQS trafficking. The supernatant of *P. aeruginosa* cultures includes large quantities of PQS, but not in a “free” soluble form. Alternatively, the highly hydrophobic PQS is encased into OMVs that traffic it within a population (Mashburn and Whiteley, 2005). Surprisingly, the same team also discovered that PQS adds to the production of OMVs by incorporating it into the outer membrane and prompting membrane curvature; thus it is also an integral membrane component (Mashburn and Whiteley, 2005; Mashburn-Warren et al., 2008). PQS beings the emergence of OMVs, and about 80% of the total PQS generated by *P. aeruginosa* PA14 is kept within vesicles (Mashburn and Whiteley, 2005). The PQS kept within these vesicles is biologically active and can reinstate the generation of virulence factors in a PQS-deficient mutant that is independent of the vesicles (Mashburn and Whiteley, 2005; Bala et al., 2015). PQS biosynthetic mutants produced remarkably reduced numbers of OMVs (Mashburn and Whiteley, 2005; Bala et al., 2015), especially late in the growth phase, when PQS would normally be present (Tashiro et al., 2010b; Macdonald and Kuehn, 2013). Compounds that halt PQS generation, including indole and its derivatives, lower OMV emergence, likely since there is smaller amount of PQS accessible to prompt vesicle establishment (Tashiro et al., 2010c). Further, exogenous PQS reinstates OMV generation in a mutant without the PQS receptor and in PQS-null cells in which protein synthesis is halted by antibiotic therapy (Mashburn and Whiteley, 2005). PQS is not synthesized under anoxic conditions, as the last step of its biosynthesis needs oxygen, and *P. aeruginosa* continuously shows lowered OMV generation when grown under anoxic conditions (Sabra et al., 2003; Schertzer et al., 2010), additionally reinforcing the notion that OMV generation is significantly lowered without PQS (Toyofuku et al., 2008; Schertzer et al., 2010). These discoveries suggest that PQS-induced OMV generation does not happen via a signaling mechanism or via the induction of a cascade, including *de novo* protein synthesis, but this appears to be a spontaneous process. Exogenous PQS has been shown to also enhance OMV production by other bacteria (Mashburn-Warren et al., 2008; Tashiro et al., 2010a). A current evaluation documented that a peptidoglycan (PG)-associated outer membrane protein, OprF, impacts OMV generation by regulating PQS generation, additionally suggesting the significance of PQS in OMV generation (Wessel et al., 2013). From these first observations, Schertzer and Whiteley suggested an OMV biogenesis model according to the biophysical impacts of PQS on the outer membrane (Schertzer and Whiteley, 2012).

Schertzer and Whiteley (2012) detailed a bilayer-coupled model through which PQS prompts OMV emergence by interacting with the acyl chains and 4′-phosphates of bacterial lipopolysaccharide (LPS) molecules and integrates them into the outer leaflet of the outer membrane, causing the enlargement of the membrane, curvature, and the eventual liberation of OMVs. PQS might also impact the 4′-phosphate via its interaction with divalent cations (Mg^2+^ and Ca^2+^) in the LPS leaflet of the outer membrane (Mashburn-Warren and Whiteley, 2006; Mashburn-Warren et al., 2008). Divalent cations secure the gram-negative outer membrane by establishing salt bridges between negatively charged phosphates of nearby LPS molecules. PQS isolates Mg^2+^ and Ca^2+^, thus impacting the movement of the 4′-phosphate of LPS (Mashburn-Warren and Whiteley, 2006; Mashburn-Warren et al., 2008). OMVs can unite with the outer membrane of neighboring recipient bacteria, where they unload their haul, which may be PQS as well as proteins, lipids, nucleic acids, and other small molecules (Jan, 2017). This model details a general mechanism through which *P. aeruginosa* might regulate OMV emergence without external stresses. Nevertheless, some other studies have demonstrated that PQS is not necessary for OMV generation in planktonic cultures under stressed or unstressed conditions (Tashiro et al., 2009; Macdonald and Kuehn, 2013; Toyofuku et al., 2014; Turnbull et al., 2016). However, a study by Florez et al. (2017) may help to solve this contradiction (Figure 1). They examined the mechanisms beneath the biogenesis of OMVs in *P. aeruginosa* without external stresses and discovered that the export of PQS induces OMV biogenesis (Macdonald and Kuehn, 2013; Florez et al., 2017). They initially hypothesized that PQS had to be moved out of the cell (via a not-yet-determined export mechanism) to prompt OMV biogenesis. To examine this hypothesis and verify that PQS export has a pivotal part in the generation of OMVs, they initially examined the generation and export of PQS and the generation of OMVs in laboratory-adapted and clinical strains of *P. aeruginosa*. They discovered significant differences in PQS export that were strongly correlated with OMV emergence. Subcellular fractionation demonstrated that poor OMV producers have significantly varying distributions of PQS among membrane compartments in contrast to strong OMV producers. In poor OMV producers that did not effectively export PQS, most of the PQS was limited to the inner membrane, while just a small amount of PQS was discovered in the inner membrane of strong OMV producers. In spite of these variations, both types of OMV producers had similar growth rates and generated similar amounts of PQS, indicating that the spatial distribution of PQS established the amount of OMVs that were generated. The researchers additionally revealed that the membrane distribution of PQS for a single strain is stable over time, but it could be changed by growth in various media, with a corresponding change in OMV generation (Florez et al., 2017). This evaluation demonstrated an immediate correlation among PQS membrane distribution and OMV biogenesis, and that by regulating the localization of PQS, researchers can control OMV production (Florez et al., 2017).

## PQS modulates host immune responses

Evaluations of PQS in *P. aeruginosa* pathogenesis have primarily zoned in on its part in modulating the generation of virulence factors. PQS may have direct effects on host cells by exerting host immunomodulatory activities (Figure 1; Hooi et al., 2004; Sadikot et al., 2005; Wu et al., 2005; Skindersoe et al., 2009; Hansch et al., 2014). With J774A.1 macrophages, PQS was revealed to regulate the expression of several genes implicated in the immune reaction and cytokine generation (Kim et al., 2010a). Not long ago, Abdalla and co-workers demonstrated that PQS prompts ROS generation *in vitro* in lung epithelial cells and hinders heme oxygenase-1 (HO-1) protein generation in lung cell lines, the latter probably through the impediment of the NrF2 pathway (Abdalla et al., 2017). PQS has also been revealed to subdue cell proliferation and interleukin-2 liberation in human peripheral blood mononuclear cells (hPBMCs) stimulated with a panactivating lectin (concanavalin A) (Hooi et al., 2004). In addition, PQS can prompt tumor necrosis factor-α liberation from hPBMCs after stimulation with LPS at concentrations over 10 μM (Hooi et al., 2004). *In vitro*, PQS hinders the generation of interleukin-12 from LPS-stimulated bone marrow-derived dendritic cells, shows a reduced capability to prompt T-cell proliferation, and lowers the antibacterial activity of the adaptive immune defense (Skindersoe et al., 2009). Further, PQS and HHQ down-regulate host innate immune systems via hindering the nuclear transcription factor-κB and hypoxia-inducible factor 1 (HIF-1) signaling pathways. This impact can be completed with cell-free extracts from cultures of wild-type *P. aeruginosa*, but not from cultures of correlating mutant derivatives (Kim et al., 2010b; Legendre et al., 2012). PQS and HHQ also activate airway epithelial bitter taste receptors (taste family 2 bitter receptor proteins; T2Rs) and stimulate T2R-mediated immune responses (Freund et al., 2018). A recent study showed that PQS stimulates neutrophil chemotaxis via activation of the MAPkinase p38, whereas PQS neither enhances the bactericidal activity of polymorphonuclear neutrophils nor induces apoptosis (Hansch et al., 2014). Altogether, these discoveries offer evidence that PQS has a pivotal part in the dysregulation of the host immune reaction throughout infection. Therefore, PQS may offer *P. aeruginosa* with another approach for bacterial survival through obstructing different host biological functions.

## Conclusion

The studies reviewed here showed that PQS, aside from its role as a QS signaling molecule, mediates iron acquisition, cytotoxicity and OMV biogenesis, and exerts host immunomodulatory activities (Figure 1). It is interesting to note that the functions of PQS usually act in a cooperative manner, providing it with an immediate part in community protection and nutrient scavenging. PQS generation may alter the entire architecture of the bacterial population, improving its fitness in several environments and causing resistance to environmental stress. Furthermore, we speculate that there are still PQS functions that remain unknown and that more research is warranted for exploring new facets of signaling, as well as non-signaling roles, of PQS for its potential application in developing modern biotechniques and treating infectious diseases.

## Author contributions

JL prepared the first draft of the manuscript and Figure 1; JL, JC, YW, and XS participated in discussion, revision and finalization of the manuscript.

### Conflict of interest statement

The authors declare that the research was conducted in the absence of any commercial or financial relationships that could be construed as a potential conflict of interest.
